# Child maltreatment, migration and risk of first-episode psychosis: results from the multinational EU-GEI study

**DOI:** 10.1017/S003329172200335X

**Published:** 2023-10

**Authors:** Giuseppe D'Andrea, Jatin Lal, Sarah Tosato, Charlotte Gayer-Anderson, Hannah E. Jongsma, Simona A. Stilo, Els van der Ven, Diego Quattrone, Eva Velthorst, Domenico Berardi, Paulo Rossi Menezes, Celso Arango, Mara Parellada, Antonio Lasalvia, Caterina La Cascia, Laura Ferraro, Daniele La Barbera, Lucia Sideli, Julio Bobes, Miguel Bernardo, Julio Sanjuán, Jose Luis Santos, Manuel Arrojo, Cristina Marta Del-Ben, Giada Tripoli, Pierre-Michel Llorca, Lieuwe de Haan, Jean-Paul Selten, Andrea Tortelli, Andrei Szöke, Roberto Muratori, Bart P. Rutten, Jim van Os, Peter B. Jones, James B. Kirkbride, Robin M. Murray, Marta di Forti, Ilaria Tarricone, Craig Morgan

**Affiliations:** 1 Department of Medical and Surgical Sciences, Bologna Transcultural Psychosomatic Team (BoTPT), University of Bologna, Bologna, Italy; 2 Community Mental Health Center of Sassuolo, Department of Mental Health and Drug Abuse, AUSL Modena, Modena, Italy; 3 Section of Psychiatry, Department of Neuroscience, Biomedicine and Movement, University of Verona, Piazzale L.A. Scuro 10, 37134, Verona, Italy; 4 ESRC Center for Society and Mental Health, Institute of Psychiatry, Psychology, and Neuroscience, King's College London, London, UK; 5 Centre for Transcultural Psychiatry ‘Veldzicht’, Balkbrug, The Netherlands; 6 University Centre for Psychiatry, University Medical Centre Groningen, Groningen, The Netherlands; 7 Department of Mental Health and Addiction Services, ASP Crotone, Crotone, Italy; 8 Department of Psychosis Studies, Institute of Psychiatry, Psychology and Neuroscience, King's College London, London, UK; 9 Department of Clinical, Neuro- and Developmental Psychology, Vrije Universiteit Amsterdam, Amsterdam, Netherlands; 10 Social, Genetic and Developmental Psychiatry Centre, Institute of Psychiatry, Psychology and Neuroscience, King's College London, London, SE5 8AF, UK; 11 Department of Psychiatry, Early Psychosis Section, Academic Medical Centre, University of Amsterdam, Amsterdam, The Netherlands; 12 Department of Psychiatry, Icahn School of Medicine at Mount Sinai, New York, USA; 13 Department of Biomedical and Neuromotor Sciences (DIBINEM), Section of Psychiatry, University of Bologna, Bologna, Italy; 14 University Hospital, Section of Epidemiology, University of São Paulo, São Paulo, Brazil; 15 Department of Child and Adolescent Psychiatry, Institute of Psychiatry and Mental Health, Hospital General Universitario Gregorio Marañón, IiSGM, School of Medicine, Universidad Complutense, CIBERSAM, Centro de Investigación Biomédica en Red de Salud Mental, Madrid, Spain; 16 Department of Biomedicine, Neuroscience and Advanced Diagnostics (BiND), Psychiatry Section, University of Palermo, Palermo, Italy; 17 Department of Human Science, LUMSA University, Rome, Italy; 18 Department of Medicine, Psychiatry Area, School of Medicine, Universidad de Oviedo, ISPA, Ineuropa, CIBERSAM, Oviedo, Spain; 19 Barcelona Clinic Schizophrenia Unit, Department of Medicine, Neuroscience Institute, Hospital Clinic, University of Barcelona, Institut d'Investigacions Biomèdiques August Pi i Sunyer, Centro de Investigación Biomédica en Red de Salud Mental, Barcelona, Spain; 20 Department of Psychiatry, School of Medicine, Universidad de Valencia, Centro de Investigación Biomédica en Red de Salud Mental, Valencia, Spain; 21 Department of Psychiatry, Servicio de Psiquiatría Hospital “Virgen de la Luz”, Cuenca, Spain; 22 Department of Psychiatry, Psychiatric Genetic Group, Instituto de Investigación Sanitaria de Santiago de Compostela, Complejo Hospitalario Universitario de Santiago de Compostela, Santiago, Spain; 23 Neuroscience and Behavior Department, Ribeirão Preto Medical School, University of São Paulo, São Paulo, Brazil; 24 Department of Health Promotion, Mother and Child Care, Internal Medicine and Medical Specialties, University of Palermo, Piazza delle Cliniche, 290127 Palermo, Italy; 25 Université Clermont Auvergne, EA 7280 Npsydo, Clermont-Ferrand, France; 26 Department of Psychiatry, Amsterdam UMC, Amsterdam, The Netherlands; 27 School for Mental Health and Neuroscience, University of Maastricht, Maastricht, The Netherlands; 28 Etablissement Public de Santé Maison Blanche, Paris, France; 29 Univ Paris Est Creteil, INSERM, IMRB, AP-HP, Hôpitaux Universitaires « H. Mondor », DMU IMPACT, Fondation Fondamental, F-94010 Créteil, France; 30 Department of Mental Health and Pathological Addiction, Local Health Authority, Bologna, Italy; 31 Department of Psychiatry and Neuropsychology, School for Mental Health and Neuroscience, Maastricht University Medical Centre, Maastricht, The Netherlands; 32 Department of Psychiatry, University of Cambridge, Cambridge, UK; 33 PsyLife Group, Division of Psychiatry, UCL, London, UK

**Keywords:** Child adversity, childhood trauma, first-episode psychosis, migrants

## Abstract

**Background:**

Child maltreatment (CM) and migrant status are independently associated with psychosis. We examined prevalence of CM by migrant status and tested whether migrant status moderated the association between CM and first-episode psychosis (FEP). We further explored whether differences in CM exposure contributed to variations in the incidence rates of FEP by migrant status.

**Methods:**

We included FEP patients aged 18–64 years in 14 European sites and recruited controls representative of the local populations. Migrant status was operationalized according to generation (first/further) and region of origin (Western/non-Western countries). The reference population was composed by individuals of host country's ethnicity. CM was assessed with Childhood Trauma Questionnaire. Prevalence ratios of CM were estimated using Poisson regression. We examined the moderation effect of migrant status on the odds of FEP by CM fitting adjusted logistic regressions with interaction terms. Finally, we calculated the population attributable fractions (PAFs) for CM by migrant status.

**Results:**

We examined 849 FEP cases and 1142 controls. CM prevalence was higher among migrants, their descendants and migrants of non-Western heritage. Migrant status, classified by generation (likelihood test ratio:χ^2^ = 11.3, *p* = 0.004) or by region of origin (likelihood test ratio:χ^2^ = 11.4, *p* = 0.003), attenuated the association between CM and FEP. PAFs for CM were higher among all migrant groups compared with the reference populations.

**Conclusions:**

The higher exposure to CM, despite a smaller effect on the odds of FEP, accounted for a greater proportion of incident FEP cases among migrants. Policies aimed at reducing CM should consider the increased vulnerability of specific subpopulations.

## Introduction

The World Health Organization (WHO) defines child maltreatment (CM) as ‘all forms of physical and/or emotional ill-treatment, sexual abuse, neglect or negligent treatment or commercial or other exploitation, resulting in actual or potential harm to child's health, survival, development or dignity in the context of a relationship of responsibility, trust or power’ (World Health Organization (WHO), 2006). Estimates of lifetime prevalence of maltreatment vary by sex and region (Moody, Cannings-John, Hood, Kemp, & Robling, 2018). Most studies have been conducted in the United States (US), where pooled estimates range between 8.0% and 36.5% according to sex and type of abuse (Stoltenborgh, Bakermans-Kranenburg, Alink, & van Ijzendoorn, 2015). There is some evidence that the prevalence of CM varies by social class (or income levels) and ethnicity (Pelton, 2015; Sedlak et al., 2010). The fourth National Incidence Study of Child Abuse and Neglect (NIS-4) in the US reported that risk of maltreatment was 5.8-times higher for children who grew up in poverty and nearly 2 times higher for those of black ethnicity (Sedlak et al., 2010). However, this report did not consider socioeconomic status (SES), introducing a potential bias in the analyses, given the high degree of economic stratification of the US and its implications (Adler & Rehkopf, 2008; Braveman, Cubbin, Egerter, Williams, & Pamuk, 2010; Kawachi, Daniels, & Robinson, 2005). In fact, more recent studies have shown that, when accounting for SES, differences in the estimates of CM between ethnic groups disappear, whereas the effect of socioeconomic disadvantage remains (Cancian, Yang, & Slack, 2013; Dworsky, Courtney, & Zinn, 2007; Kim & Drake, 2018; Putnam-Hornstein, Needell, King, & Johnson-Motoyama, 2013; Slack, Lee, & Berger, 2007). That is, ethnic disparities in risk of socioeconomic disadvantage may drive the observed differences in CM. In turn, the association with socioeconomic disadvantage undoubtedly reflects the consequence of structural inequalities in the access to basic material resources. European studies, such as the Netherlands' Prevalence study of Maltreatment (NPM) (Alink, Euser, van IJzendoorn, & Bakermans-Kranenburg, 2013; Euser, van IJzendoorn, Prinzie, & Bakermans-Kranenburg, 2011; van Berkel, Prevoo, Linting, Pannebakker, & Alink, 2020), yielded similar results, showing that migrants and their descendants from different ethnic backgrounds had from around a 2- to 3- fold increased likelihood of maltreatment compared with Dutch (van Berkel et al., 2020). These differences were partly explained by measures of social disadvantage (Alink et al., 2013).

CM is linked to subsequent development of physical and mental disorders (World Health Organization (WHO), 2006). Several meta-analyses (Arango et al., 2021; Varese et al., 2012) have shown that the odds of developing psychosis among those exposed to maltreatment is around 2–3 times the odds among those not exposed. The effect of CM on odds of psychosis is cumulative and affected by type, age of exposure, and severity (Morgan et al., 2020).

Psychosis incidence is higher among migrants and their descendants (Morgan, Knowles, & Hutchinson, 2019; Selten, Van Der Ven, & Termorshuizen, 2019) and vary by region of origin, being higher among those from non-Western countries, and by region of resettlement, peaking in sites where incidence of psychosis is higher also for local populations (Termorshuizen et al., 2020). Morgan et al. ( 2019) have proposed a ‘socio-developmental pathway’ to psychoses, according to which greater exposure to social adversities across the life course accounts for the elevated rates of psychosis in migrants and their descendants. In two recent studies (Gatt et al., 2020; Solà-Sales et al., 2021) the effect of traumatic events on mental health was lower among migrants compared to non-migrants, hindering at possibly greater resilience. However, none of these studies specifically looked at the risk of psychosis.

In this context, we aimed to: (1) estimate the prevalence of specific CM subtypes among migrants considering generational status and regions of origin compared with individuals of host country's ethnicity; (2) test whether migrant status moderated the association between CM and first-episode psychosis (FEP); (3) examine whether CM exposure accounted for a more sizeable proportion of incident FEP cases among migrants compared with the reference populations.

## Methods

### Study design and participants

This study is part of the European network of national schizophrenia networks studying Gene–Environment Interactions (EU-GEI) project (http://www.eu-gei.eu) (Gayer-Anderson et al., 2020). Participants were recruited between 1 May 2010, and 1 April 2015 from sixteen centers in England, the Netherlands, Spain, France, Italy and Brazil. All persons aged 18–64 years who made contact with mental health services for a probable FEP were invited to participate (Gayer-Anderson et al., 2020). Cases were included if they met International Classification of Disease (ICD)-10 criteria for psychotic disorders (F20–33), ascertained using the Operational Criteria Checklist (OPCRIT) algorithm (Quattrone et al., 2019; Williams et al., 1996). Assessment with OPCRIT relied on semistructured clinical interviews or review of case notes and other relevant information.

In each center, we recruited population-based controls aged 18–64 years using a mixture of random and quota-sampling strategies, to maximize representativeness to the population-at-risk by age, sex and ethnicity. Quotas were derived from the most accurate local demographic data. Strategies for control recruitment included random sampling from lists of all postal addresses; stratified sampling via General Practitioners lists from randomly selected surgeries; and advertisement via multiple channels (internet, newspapers, leaflets). Individuals with a history of psychotic disorder, or taking anti-psychotic medication, were not eligible (Di Forti et al., 2019; Gayer-Anderson et al., 2020). As shown by a previous EU-GEI publication, controls were broadly representative of the population-at-risk on sex and ethnicity, but in some sites they were younger (Jongsma et al., 2020).

Ethical approval was granted in each center. All participants gave written informed consent.

### Measures

#### Outcome

Our primary outcome was case–control status, with cases defined as receiving an OPCRIT-confirmed ICD-10 diagnosis of any psychotic disorder (ICD-10 codes F20–F33).

#### Socio-demographic and migrant status

We collected data on age at first diagnosis/control recruitment, sex, ethnicity, education (years), parental social class (long-term unemployed, working class, intermediate, salariat), and personal and parental country of birth, using an amended version of the Medical Research Council Socioeconomic Schedule (Mallet, 1997). First-generation migrants were identified based on their birth country. Information on parental country of birth and site-specific ethnicity were used to identify migrants' descendants. Consistent with a previous EU-GEI publication (Termorshuizen et al., 2020), migrants and their descendants were also grouped according to personal or parental regions of origin into individuals from Western and non-Western countries. Western countries included Europe, US, Canada, Australia, New Zealand, and countries of the former Soviet Union with a predominantly Christian religion. Non-Western countries included the following areas: Middle East & The Maghreb; sub-Saharan Africa; Asia; Latin America; The Caribbean Islands and the French overseas departments. We refer to the prior paper for more detailed information (Termorshuizen et al., 2020). In each site, the reference population was composed of individuals of host country's ethnicity, born in the country of recruitment and with both parents born in that same country. We excluded all individuals who could not be ascribed with certainty to any of the aforementioned groups.

#### Child maltreatment

CM was assessed using the Childhood Trauma Questionnaire (CTQ) (Bernstein et al., 2003). It consists of five items for each subtype of trauma (*emotional abuse*, *emotional neglect*, *physical abuse*, *physical neglect*, and *sexual abuse*). Every item is rated on a 5-point Likert-type scale (1 = never true, 5 = very often true) and allows for computation of both mean scores for each type of abuse (range 5–25) and categorical scores, with fixed threshold values for each subdomain (none to minimal, slight to moderate, moderate to severe, severe to extreme) (Bernstein et al., 2003). Walker et al. (Walker et al. 1999) have proposed another procedure for the CTQ rating and, based on a comparison between self-reported CTQ scores and expert ratings of structured clinical interviews, determined threshold scores with both sensitivity and specificity ⩾0.85 (online Supplementary Materials). Walker et al.'s (Walker et al., 1999) method provides dichotomized measures of *emotional abuse*, *emotional neglect*, *physical abuse*, *physical neglect*, and *sexual abuse*. Conform previous analyses on the EU-GEI dataset (Sideli et al., 2022), CM exposure was operationalized as a dichotomous variable using the 80th percentile of the mean CTQ score in the control group as cut-off value. For the moderation analyses, we used the mean of the CTQ subscales scores (range: 5–25) as an overall maltreatment score.

#### Other exposures

Parental history of psychosis and mental illness was recorded using the Family Interview for Genetic Studies questionnaire (Maxwell, 1992). Current cannabis use (no/yes) was derived from a modified version of the Cannabis Experience Questionnaire (Di Forti et al., 2009).

### Missing data

The proportion of participants with missing data ranged from none on sex and country of recruitment to 269 (13.5%) on parental psychosis. Complete data were available for 1502 participants (75.4%). Missing data were handled by multiple imputation (details in online Supplementary Materials).

### Statistical analyses

Differences in exposures and covariates distribution by case–control status on the whole sample and by migrant status were conducted using χ^2^ tests for categorical variables and student's *t* tests for continuous variables.

The analyses on prevalence of CM by migrant status were conducted only on controls, who were broadly representative of the population at risk within each catchment area (Gayer-Anderson et al., 2020). We estimated the prevalence ratios (PR) of each type of CM [dichotomized yes/no (Walker et al., 1999)] by migrant generational status and by migrants' region of origin (with white majority as reference population) using Poisson regression with robust variance (Barros & Hirakata, 2003; Santos et al., 2008). PRs were adjusted for age, sex, parental social class, educational attainment, parental mental illness, and site of recruitment. Models' goodness-of-fit was tested with χ^2^d tests.

To test our second hypothesis, we used a mixed-effects logistic regression with random intercepts at the site level to account for clustering by recruitment site (*n* = 14) while estimating odds ratios (OR) for case–control status from the following independent variables: CM (CTQ mean score), migrant status, age, sex, education, parental psychosis, cannabis use, and parental social class at birth. We used polychoric correlations to describe associations between confounders and exposures and estimated Variance Inflation Factor to further check for multicollinearity (online Supplementary Materials). We finally introduced a multiplicative interaction term to the model between migrant status and CTQ mean score. The CTQ score was standardized for the purpose of interaction analysis. We performed a likelihood ratio test to assess whether the addition of the interaction term improved the model (Aiken, West, & Reno, 1991). Then, we estimated the stratified OR for each migrant group from the model with the interaction term by combining the coefficients. Finally, we used the command ‘*margins*’ in Stata 17 to estimate the adjusted predicted probabilities with 95% CI, by migrant status, of being a case at fixed values of standardized mean CTQ using the marginal standardization method (Muller & MacLehose, 2014). Predicted probabilities at the selected values of CTQ along with standard errors and *p* values were reported in online Supplementary Materials and were plotted for better reporting the interaction effect. Residual diagnostics of the mixed models were performed using the R package ‘DHARMa’ (Hartig, 2022) (online Supplementary Materials). We controlled the false discovery rate using the Benjamini-Hochberg procedure (Benjamini & Hochberg, 1995), tolerating a 10% false discovery rate. Both unadjusted and adjusted *p* values are reported.

Lastly, we refitted the same mixed-effect logistic regression models substituting the total CTQ score for a dichotomous variable obtained using the 80th percentile of CTQ total score of controls as cut-off value (Sideli et al., 2022). We used the command ‘punafcc’ in Stata 17 to calculate the population attributable fraction (PAF) for CM exposure by migrant status. Assuming causality, PAF represents the estimated fraction of FEP cases that would not have occurred if there had been no exposure to CM.

Sensitivity analyses were conducted on complete cases only for the main outcomes (online Supplementary Materials).

Analyses were performed using Rstudio R version 3.6.3 (Rstudio Team (2020). *Rstudio: Integrated Development for R. Rstudio*, PBC, Boston, MA URL http://www.rstudio.com/) and Stata 17 (StataCorp. 2021. *Stata Statistical Software: Release 17*. College Station, TX: StataCorp LLC). All analyses were conducted following imputation of the missing values.

## Results

Between 2010 and 2015, 1130 cases and 1497 controls were recruited and assessed across 17 sites in 6 countries (UK, The Netherlands, France, Spain, Italy, and Brazil). We excluded Brazil (*n* = 494) due to differences in the context and in the migration patterns with respect to European countries. We also excluded the French site of Maison-Blanche (*n* = 36) as no control was recruited there, and the Spanish site of Santiago (*n* = 64) due to the underrepresentation of migrants (*n* = 2, 3.2%). For 40 participants, it was not possible to determine their region of origin or migrant generational status due to missing data on either personal/parental country of birth or ethnicity. This resulted in an analysis sample of 1991 individuals (849 cases and 1142 controls).

### Characteristics of the case–control sample

The distribution of exposures and covariates in the case–control sample is described in Table 1. Cases were more likely than controls to be men (χ^2^ = 46.7; *p* < 0.001) and younger (*t* = 10.2; *p* < 0.001). They also received less education (*t* = 9.4; *p* < 0.001) and more often reported lower parental SES (χ^2^ = 13.3; *p* = 0.004). Parental mental illness (χ^2^ = 24.3; *p* < 0.001) and psychosis (χ^2^ = 33.8; *p* < 0.001) were more frequent among cases. The latter were more likely to report use of cannabis (χ^2^ = 41.4; *p* < 0.001) and scored higher than controls on the total CTQ score (*t* = −14.5; *p* < 0.001). Finally, *emotional abuse* (χ^2^ = 101.2; *p* < 0.001), *emotional neglect* (χ^2^ = 71.8; *p* < 0.001), *physical abuse* (χ^2^ = 95.4; *p* < 0.001), *physical neglect* (χ^2^ = 129.2; *p* < 0.001), and *sexual abuse* (χ^2^ = 47.1; *p* < 0.001), were more frequent among cases than among controls. Distribution of exposures and covariates among cases and controls by migrant generational status and region of origin is detailed in online Supplementary Materials (Tables S3, S4).

**Table 1.** Distribution of exposures and covariates in the case–control sample

		Total sample (*N* = 1991)		
		Controls (*N* = 1142)	Cases (*N* = 849)	χ^2^/*t* (*p*)
Gender	Males	604 (52.9%)	318 (37.5%)	**46.7 (*p* < 0.001)**^a^
	Females	538 (47.1%)	531 (62.5%)	
Age	Mean (s.d.)	36.6 (13.1)	31.1 (10.4)	**10.2 (<0.001)**^b^
Migrant generational status	Reference population	769 (67.3%)	478 (56.3%)	**28.7 (<0.001)**^a^
	First generation	214 (18.7%)	237 (27.9%)	
	Further generation	159 (13.9%)	134 (15.8%)	
Migrant group	Reference population	769 (67.3%)	478 (56.3%)	**35.0(<0.001)**^a^
	Western migrants	131 (11.5%)	92 (10.8%)	
	Non-western migrants	242 (21.2%)	279 (32.9%)	
Education (years)	Mean (s.d.)	15.4 (3.9)	13.8 (3.9)	**9.4(<0.001)**^b^
Parental social class	Professional	389 (34.1%)	247 (29.1%)	**13.3(0.004)**^a^
	Intermediate	277 (24.3%)	196 (23.1%)	
	Working class	468 (41.0%)	389 (45.8%)	
	Long-term unemployed	8 (0.7%)	17 (2.0%)	
Parental psychosis	No	1123 (98.3%)	792 (93.3%)	**33.8(<0.001)**^a^
	Yes	19 (1.7%)	57 (6.7%)	
Parental mental illness	No	885 (77.5%)	574 (67.6%)	**24.3(<0.001)**^a^
	Yes	257 (22.5%)	275 (32.4%)	
Cannabis use	No	1006 (88.1%)	656 (77.3%)	**41.4(<0.001)**^a^
	Yes	136 (11.9%)	193 (22.7%)	
Maltreatment exposure				
Child maltreatment†	No	936 (82.0)	475 (55.9)	**159.6(<0.001)**^a^
	Yes	206 (18.0)	374 (44.1)	
Emotional abuse	<10	953 (83.5%)	541 (63.7%)	**101.2(<0.001)**^a^
	⩾10	189 (16.5%)	308 (36.3%)	
Emotional neglect	<15	999 (87.5%)	615 (72.4%)	**71.8(<0.001)**^a^
	⩾15	143 (12.5%)	234 (27.6%)	
Physical abuse	<8	1018 (89.1%)	612 (72.1%)	**95.4(<0.001)**^a^
	⩾8	124 (10.9%)	237 (27.9%)	
Physical neglect	<8	891 (78.0%)	458 (53.9%)	**129.2(<0.001)**^a^
	⩾8	251 (22.0%)	391 (46.1%)	
Sexual abuse	<8	1058 (92.6%)	702 (82.7%)	**47.1(<0.001)**^a^
	⩾8	84 (7.4%)	147 (17.3%)	

s.d., standard deviation.

a Pearson's χ^2^ test.

b *t* student's test

^†^Defined as mean CTQ > 80th percentile of the control group^.^

### Prevalence of the different forms of CM among migrants and descendants compared with reference population (controls only analyses)

In controls, the distribution of CM exposure differed significantly among the study groups, with both first-generation migrants (*N* = 56, 26.2%) and their descendants (*N* = 36, 22.6%) being more likely to report CM compared with the reference populations (*N* = 114, 14.8%) (χ^2^ = 17.2, *p* < 0.001). Similarly, a considerably greater proportion of migrants from both non-Western (*N* = 63, 26.0%) and Western countries (*N* = 29, 22.1%) was exposed to CM compared with the reference populations (*N* = 114, 14.8%) (χ^2^ = 17.2, *p* < 0.001).

Unadjusted and adjusted PR of the measured subtypes of CM by migrant generational status and region of origin are presented in Tables 2 and 3 (Tables 2, 3). The prevalence of *emotional abuse* (aPR = 1.47, 95% CI 1.06–2.04), *physical abuse* (aPR = 3.28, 95% CI 2.18–4.94), *physical neglect* (aPR = 1.96, 95% CI 1.52–2.53), and *sexual abuse* (aPR = 1.97, 95% CI 1.17–3.33) were higher among first-generation migrants compared with the reference populations. Children of migrants reported greater PRs of *physical abuse* (aPR = 2.50, 95% CI 1.61–3.88) and *sexual abuse* (aPR = 1.97, 95% CI 1.17–3.33) than the reference populations. Considering region of origin, migrants and their descendants from non-Western countries showed higher prevalence of *emotional abuse* (aPR = 1.50, 95% CI 1.09–2.06), *physical abuse* (aPR = 3.57, 95% CI 2.38–5.36), *physical neglect* (aPR = 1.83, 95% CI 1.40–2.40), and *sexual abuse* (aPR = 2.44, 95% CI 1.50–3.97) compared with the reference populations. People with a Western migrant heritage only had a higher prevalence of *physical abuse* (aPR = 2.08, 95% CI 1.24–3.48) in relation to the reference group.

**Table 2.** Unadjusted and adjusted PRs of CM subtypes by migrant generational status

	Reference (*N* = 769)		First generations (*N* = 214)			Further generations (*N* = 159)		
	*N* (%)	PR (95% CI)	*N* (%)	PR (95% CI)	aPR (95% CI)	*N* (%)	PR (95% CI)	aPR (95% CI)
EA	108 (14.0%)	Ref	47 (22.0%)	**1.56** (**1.15–2.13)**	**1.47** (**1.06–2.04)**	34 (21.4%)	**1.52** (**1.08–2.15)**	1.38 (0.97–1.95)
EN	91 (11.8%)	Ref	32 (15.0%)	1.26 (0.87–1.84)	1.38 (0.94–2.05)	20 (12.6%)	1.06 (0.68–1.67)	1.14 (0.71–1.83)
PA	46 (6.0%)	Ref	48 (22.4%)	**3.75** (**2.58–5.46)**	**3.28** (**2.18–4.94)**	30 (18.9%)	**3.15** (**2.06–4.83)**	**2.50** (**1.61–3.88)**
PN	148 (19.2%)	Ref	71 (33.2%)	**1.72** (**1.36–2.19)**	**1.96** (**1.52–2.53)**	32 (20.1%)	1.05 (0.74–1.47)	1.16 (0.82–1.65)
SA	45 (5.9%)	Ref	22 (10.3%)	**1.76** (**1.08–2.86)**	**1.97** (**1.17–3.33)**	17 (10.7%)	**1.83** (**1.07–3.11)**	**1.98** (**1.15–3.40)**

PR, prevalence ratio; 95% CI, 95% confidence interval; aPR, adjusted prevalence ratio; EA, emotional abuse; EN, emotional neglect; PA, physical abuse; PN, physical neglect; SA, sexual abuse.

PRs were adjusted for age, gender, educational attainment, parental social class, parental mental illness, and site of recruitment. PRs in bold are significant at 0.05 level.

**Table 3.** Unadjusted and adjusted PRs of CM subtypes by migrants' area of origin

	Reference (*N* = 769)		Western (*N* = 214)			Non-western (*N* = 159)		
	*N* (%)	PR (95% CI)	*N* (%)	PR (95% CI)	aPR (95% CI)	*N* (%)	PR (95% CI)	aPR (95% CI)
EA	108 (14.0%)	Ref	47 (22.0%)	1.41 (0.96–2.08)	1.33 (0.91–1.94)	34 (21.4%)	**1.62** (**1.21–2.16)**	**1.50** (**1.09–2.06)**
EN	91 (11.8%)	Ref	32 (15.0%)	1.03 (0.63–1.70)	1.16 (0.71–1.90)	20 (12.6%)	1.26 (0.88–1.80)	1.36 (0.92–2.02)
PA	46 (6.0%)	Ref	48 (22.4%)	**2.30** (**1.38–3.83)**	**2.08** (**1.24–3.48)**	30 (18.9%)	**4.14** (**2.90–5.92)**	**3.57** (**2.38–5.36)**
PN	148 (19.2%)	Ref	71 (33.2%)	1.15 (0.81–1.64)	1.34 (0.94–1.91)	32 (20.1%)	**1.59** (**1.25–2.02)**	**1.83** (**1.40–2.40)**
SA	45 (5.9%)	Ref	22 (10.3%)	1.17 (0.59–2.34)	1.31 (0.65–2.64)	17 (10.7%)	**2.12** (**1.37–3.29)**	**2.44** (**1.50–3.97)**

PR, prevalence ratio; 95% CI, 95% confidence interval; aPR, adjusted prevalence ratio; EA, emotional abuse; EN, emotional neglect; PA, physical abuse; PN, physical neglect; SA, sexual abuse.

PRs were adjusted for age, gender, educational attainment, parental social class, parental mental illness, and site of recruitment. PRs in **bold** are significant at 0.05 level.

### Associations of CM with FEP in reference populations and migrant populations

Both first-generation migrants (OR 1.88, 95% CI 1.49–2.38; *p* < 0.001, *p*_adj_ < 0.001) and their descendants (OR 1.47, 95% CI 1.11–1.95; *p* = 0.008, *p*_adj_ = 0.014) had greater odds of FEP compared with the reference populations in the unadjusted mixed-effects model. Migrants and their descendants from non-Western countries also had increased odds of FEP compared with the reference population (OR 2.03, 95% CI 1.60–2.58; *p* < 0.001, *p*_adj_ < 0.001); odds were not statistically different for migrants from Western countries compared with the reference population (OR 1.24, 95% CI 0.91–1.68; *p* = 0.171, *p*_adj_ = 0.243).

**Fig. 1.**
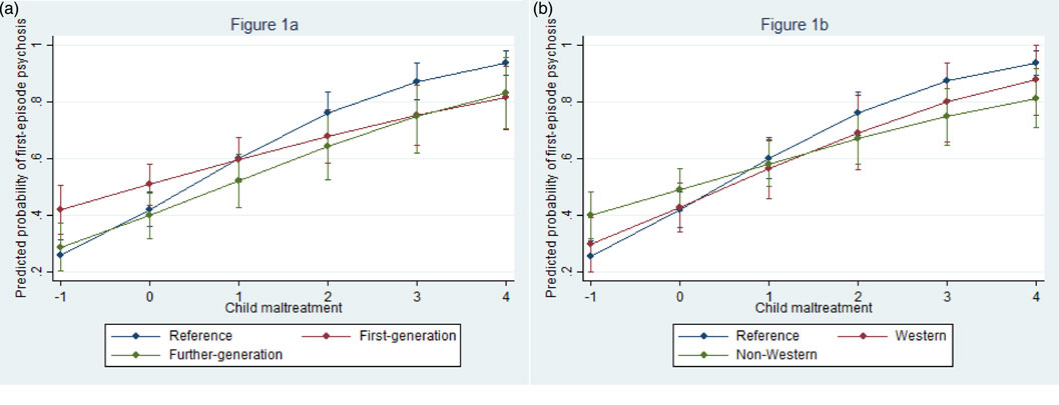
Predicted probabilities of FEP by CM and migrant status. (a) represents predicted probabilities of psychosis by CM and migrant generational status. (b) represent predicted probabilities of psychosis by CM and Western/non-Western migrant status.

In the model with migrants classified by generational status, every 1-s.d. increase in the CTQ score was associated with almost 2-fold increased odds of FEP (OR 1.98, 95% CI 1.75–2.23; *p* < 0.001, *p*_adj_ < 0.001). The addition of the interaction term ‘migrant × maltreatment’ improved the model (likelihood test ratio: χ^2^ = 11.4, *p* = 0.003, *p*_adj_ = 0.007). The stratified OR from the model with the interaction term suggested that the association between CTQ and FEP was stronger among individuals from the reference population (OR 2.42, 95% CI 2.03–2.89; *p* < 0.001, *p*_adj_ < 0.001) than among first-generation migrants (OR 1.54, 95% CI 1.26–1.89; *p* < 0.001, *p*_adj_ < 0.001) or their descendants (OR 1.83, 95% CI 1.43–2.33; *p* < 0.001, *p*_adj_ < 0.001) (Table 4). Figure 1a shows the estimated predicted probabilities of FEP among the different groups for varying scores of CTQ. For increasing values of CTQ, predicted probabilities of FEP were higher among the reference population compared with first-generation migrants or their children (Fig. 1a).

**Table 4.** Odds ratios of FEP by migrant status and migrant-CM interaction

			Unadjusted		Adjusted^a^		Migrant × CTQ^b^		PAF (95% CI)^c^
	*N* controls (%)	*N* cases (%)	OR (95% CI)	*p*	OR (95% CI)	*p*	OR (95% CI)	*p*	
By generational status									
Reference	769 (67.3%)	478 (56.3%)	Ref.	–	Ref.	–	**2.42** (**2.03–2.90)**	<0.001	27.9% (25.3–30.3%)
First generation	214 (18.7%)	237 (27.9%)	**1.88** (**1.49–2.38)**	<0.001	**1.53** (**1.17–2.01)**	0.002	**1.54** (**1.26–1.89)**	<0.001	37.3% (33.8–40.5%)
Further generation	159 (13.9%)	134 (15.8%)	**1.47** (**1.11–1.95)**	0.008	0.93 (0.67–1.29)	0.662	**1.83** (**1.43–2.33)**	<0.001	36.7% (33.4–39.9%)
By area of origin									
Reference	769 (67.3%)	478 (56.3%)	Ref.	–	Ref.	–	**2.42** (**2.03–2.89)**	<0.001	27.9 (25.4–30.3%)
Western	131 (11.5%)	92 (10.8%)	1.24 (0.91–1.68)	0.171	1.06 (0.75–1.50)	0.737	**1.95** (**1.43–2.65)**	<0.001	34.7% (31.5–37.7%)
Non-Western	242 (21.2%)	279 (32.9%)	**2.03** (**1.60–2.58)**	<0.001	**1.40** (**1.07–1.84)**	0.015	**1.57** (**1.31–1.87)**	<0.001	37.9% (34.5–41.2%)

OR, odds ratio; 95% CI, 95% confidence interval; PAF, proportion of attributable fraction.

a Odds ratios were adjusted for age, gender, educational attainment, cannabis use, parental social class, and parental psychosis.

b Odds ratios were estimated by linear combination of the coefficients from the interaction model.

c PAFs were estimated from the fully adjusted models with the interaction terms.

All models were mixed effect models accounting for clustering by site of recruitment (*n* = 14).

Similarly, in the model with migrants and their descendants classified by region of origin, the addition of the interaction term ‘migrant × maltreatment’ improved the model (likelihood test ratio: χ^2^ = 11.4, *p* = 0.003, *p*_adj_ = 0.005). The association between CM and FEP was more robust among individuals from the reference population (OR 2.42, 95% CI 2.03–2.89; *p* < 0.001, *p*_adj_ < 0.001) compared with migrants from Western (OR 1.95, 95% CI 1.43–2.65; *p* < 0.001, *p*_adj_ < 0.001) or non-Western countries (OR 1.57, 95% CI 1.31–1.87; *p* < 0.001, *p*_adj_ < 0.001) (Table 4). Predicted probabilities of FEP by migrant status and CM are plotted in Fig. 1b. The effect of higher CTQ scores on the probability of FEP was weaker among migrants and their descendants from Western and non-Western countries compared with the reference population (Fig. 1b).

### Population attributable fractions

The PAF for CM exposure in the total sample was 31.9% (95% CI 28.9–34.7%). PAF was higher for among first-generation migrants (37.3%, 95% CI 33.8–40.5%) and descendants (36.7%, 95% CI 33.4–39.9%) as well as among Western (34.7%, 95% CI 31.5–37.7%) and non-Western migrants (37.9%, 95% CI 34.5–41.2%) compared with the reference populations (27.9%, 95% CI 25.4–30.3%).

### Sensitivity analyses

Results of the analyses conducted in the complete-case sample did not differ from imputed analyses (online Supplementary Materials).

## Discussion

### Main findings

We found that migrants and their descendants and participants from non-Western countries reported greater exposure to CM specific subtypes compared with the reference population. In adjusted models, first-generation migrants and those of non-Western heritage also had about 1.5-times higher odds of FEP compared with the reference group. Migrant status moderated the association between CM and FEP by attenuating the effect of trauma on the predicted probabilities of psychosis. Finally, according to our results, CM accounted for about one third of incident FEP cases (PAF = 31.9%, 95% CI 28.9–34.7%). Estimated PAFs were higher among all migrant groups compared with the reference populations.

### Strengths and limitations

To the best of our knowledge, this is the first study to investigate the association between CM and FEP between migrants and majority populations. We used data from a large case–control study. All relevant information was collected using validated instruments and a standardized data-entry was ensured to minimize collection biases across sites. The broad range of confounders that have been considered provides more certainty that the associations we found are independent of potentially confounding effects. The control recruitment strategy was designed to obtain a sample representative of the population-at-risk, allowing us to calculate reliable estimates of the prevalence of CM in local populations. CM measures for the estimation of prevalence were operationalized using highly sensitive and specific cut-off scores which were proposed in 1999 by Walker et al. (Walker et al. 1999) and have consistently been utilized over the past years (Brühl, Kley, Grocholewski, Neuner, & Heinrichs, 2019; Khosravani, Messman-Moore, Mohammadzadeh, Ghorbani, & Amirinezhad, 2019; Renna et al., 2021; Vaskinn, Melle, Aas, & Berg, 2021). We also dichotomized the CTQ total score to calculate PAFs for each study group conform previous analyses in this sample (Sideli et al., 2022). Nevertheless, we acknowledge that dichotomization of variables is associated with increased risk of both type I and II error.

This study, however, has several limitations. First, the total EU-GEI case–control comprised over 1000 cases and 1000 controls. A sample of this size has over 90% power to detect OR of 1.5 or greater at *p* < 0.05 when the prevalence of the exposure is 15% or greater. Our analyses were conducted on a slightly smaller sample (*N* = 1991) with less power to detect relevant associations. We examined the interplay between CM and migrant status on psychosis risk. This was not a primary objective of the EU-GEI study. Nonetheless, our hypotheses, though secondary, were built on previous EU-GEI publications (Aas et al., 2021; Tarricone et al., 2021; Termorshuizen et al., 2020). The retrospective assessment of the exposures may lead to recall bias. Specifically, the CTQ relies on the subjects' recollection, and we therefore cannot exclude the possibility of recall or desirability bias entering the study; we do not know whether these effects would have been differential by migrant status. Nonetheless, evidence suggests that this limitation may not be as critical as commonly thought (Brewin, Andrews, & Gotlib, 1993). In our case, individuals with FEP were more likely to have missing data on CTQ than controls. This may have led to either underestimate or overestimate the true association between CM and FEP. However, imputation of missing values was implemented to minimize loss of precision and selection biases potentially deriving from complete-case analysis (Stekhoven & Bühlmann, 2012; Waljee et al., 2013). The reliability of retrospective reports of CM by individuals with psychosis has been questioned as recall of events could be potentially biased by features of the disease such as cognitive impairment or delusional beliefs (Susser & Widom, 2012). However, previous research showed that memories of CM seem reasonably accurate and stable in individuals with psychosis (Fisher et al., 2011) and equally biased independently of psychiatric status (Fergusson, Horwood, & Woodward, 2000). Importantly, the CTQ does not provide any information on the perpetrators, nor about the context where the maltreatment took place or its duration over time. Finally, it is possible that the experiences captured among migrants differ from those captured among the reference population (e.g. sexual violence in the context of war and ‘neglect’ in the context of social deprivation). Another limitation is that this study did not consider the age at exposure to the adversity. Evidence from neuroimaging suggest that abuse could have a more ‘detrimental’ effect if perpetrated within a specific age range (Pechtel, Lyons-Ruth, Anderson, & Teicher, 2014). In conclusion, study results need replication on prospective studies with larger sample size.

### Comparison with previous evidence: prevalence of maltreatment by migrant status

Migrants reported greater exposure to cumulative trauma than the reference population, independently of generational status. Regarding the specific maltreatment subtypes, rates were 1.5 to 3.0 times higher for *emotional abuse*, *physical abuse*, *physical neglect*, and *sexual abuse* among first-generation migrants, and 2.0 to 2.5-times higher for *sexual abuse* and *physical abuse* respectively among second-generation migrants in the fully adjusted models. We also found higher PRs of almost all maltreatment subtypes, except for *emotional neglect*, for migrants and their descendants from non-Western countries, while Western migrants only showed a 2-times higher exposure to *physical abuse* compared with the reference population. Previous studies comparing the prevalence of CM in migrants with non-migrants have reported mixed, and partially contradictory findings. For example, in a study conducted in the US (Vaughn et al., 2017), both the reference population and second-generation migrants were more likely than first-generation migrants to report any form of abuse, but less likely to report any form of neglect. Other research from the same country, found that children from ethnic minority backgrounds reported more maltreatment (Sedlak et al., 2010). Among European studies, papers from the NPM (Alink et al., 2013; Euser et al., 2011; van Berkel et al., 2020) of youth consistently replicated the finding of an increased risk of maltreatment among migrants compared with Dutch majority, with the last study reporting a RR of 3.41 (95% CI 2.47–4.70) for first- and of 1.93 (95% CI 1.52–2.45) for second-generation migrants (van Berkel et al., 2020). In the group of migrants which the authors defined as ‘traditional’ based on a relatively longer migration history in the Netherlands (i.e. Turkish, Moroccan, Surinamese, and Antillean), the higher risk of maltreatment compared with the Dutch majority reference group was partially explained by socioeconomic and family factors (i.e. SES, parental education, and single parenthood) (Alink et al., 2013). In our study, the addition of measures of socioeconomic and family factors (i.e. parental SES and parental mental illness) to the models attenuated the PRs of some CM subtypes, such as *emotional abuse* and *physical abuse*. Measures of social deprivation that we have not captured (e.g. household-related variables, income, or home ownership data) may explain the higher levels of maltreatment. We also did not assess specific factors within the family, including single/step-parenthood, family separation, parental stress, or traumatization of migrant families in their country of origin. The latter is particularly important, as post-traumatic stress disorder and traumatic experiences are predictors of CM (Mbagaya, Oburu, & Bakermans-Kranenburg, 2013; Montgomery, Just-Østergaard, & Jervelund, 2019), and can be up to 10 times more common in migrants compared with the general population (Fazel, Wheeler, & Danesh, 2005). Together, this research suggests we need to do more to identify the upstream causes of the causes; that is what factors explain why migrant and their descendants report higher levels of maltreatment. It is these proximal causes that may be susceptible to interventions.

### Comparison with previous evidence: FEP risk associated with maltreatment by migrant status

Consistent with the existing literature (Morgan & Gayer-Anderson, 2016; Morgan et al., 2020; Varese et al., 2012), we found that experiences of CM increased the odds of adulthood FEP in all groups. Migrant status attenuated this association. This is in line with two recent studies which found that, despite a greater exposure to traumatic events, migrants showed greater resilience and a smaller detrimental effect of trauma on their mental health, compared with the reference populations (Gatt et al., 2020; Solà-Sales et al., 2021).

Finally, to our knowledge no prior study has examined PAFs for CM according to migrant status. Our results on the overall sample (PAF = 32.8%, 95% CI 29.4–35.6%), though, are consistent with the evidence provided by previous meta-analyses (Dragioti et al., 2022; Varese et al., 2012). The finding that CM accounts for a greater PAF in migrants and their descendants compared with non-migrants aligns with the hypothesis of a ‘socio-developmental pathway’ to psychosis (Morgan et al., 2019).

### Relevance and implications

CM is a widespread public health issue which needs global and effective interventions. Our results confirm the well-established association between CM and psychosis. Migrants and their descendants in our study had greater prevalence of most maltreatment subtypes. Despite greater exposure to CM, the association between maltreatment and psychosis was stronger for those from the reference population compared with migrants and their descendants. Migrants experience 2 to 3-times higher rates of psychosis compared with the non-migrant population (Selten et al., 2019). Reasons for the increased rates are yet to be completely understood. Our results, however, suggest that, despite a marginally smaller effect on the odds of FEP, the considerably greater exposure to CM among migrants and their children accounted for a more sizable proportion of incident FEP cases. Therefore, public health policies aimed at addressing the issue of CM by the means of adequate interventions for each level of prevention should consider the greater vulnerability of some groups, such as migrants and their descendants, and ensure that such programs are accessible to these population sections. Exposure to CM is not the sole explanation for the higher psychosis risk among migrants and their descendants. The ‘social defeat’ hypothesis (Selten & Cantor-Graae, 2005) posits that the prolonged experience of exclusion from the majority groups increases the risk of psychosis by sensitizing the dopamine pathway. Further evidence on the contribution of cumulative exposure to psycho-social adversities derives from previous reports on this issue from the EU-GEI study (Jongsma et al., 2020; Misra et al., 2021; Tarricone et al., 2021). More studies are needed to better understand the association between migrant status and psychosis focusing on putative causal factors.

## Supplementary material

For supplementary material accompanying this paper visit https://doi.org/10.1017/S003329172200335X. click here to view supplementary material
