# Supplementary material for: Child maltreatment, migration and risk of first-episode psychosis: results from the multinational EU-GEI study
Source: Psychol Med. 2022 Oct 28;53(13):6150–60. doi: 10.1017/S003329172200335X (PMC10520604; doi:10.1017/S003329172200335X)
Supplement: Supplementary file 1 [file S003329172200335Xsup001.docx]

Table of contents

[**Missing data** 2](#_Toc112596662)

[***Table S1.* Distribution of exposures and covariates in the non-imputed case-control sample.** 3](#_Toc112596663)

[***Table S3.* Distribution of exposures and covariates by case-control and migrant generational status.** 5](#_Toc112596664)

[***Table S4.* Distribution exposures and covariates by case-control and migrant group.** 7](#_Toc112596665)

[**Correlations between child maltreatment subtypes** 9](#_Toc112596666)

[***Table S5.* Correlation matrix of child maltreatment subtypes.** 9](#_Toc112596667)

[**Correlations between exposures and confounders** 9](#_Toc112596668)

[***Table S6.* Correlation matrix of predictors included in the model with migrants defined based on generational status.** 9](#_Toc112596669)

[***Table S7.* Correlation matrix of predictors included in the model with migrants defined based on area of origin.** 9](#_Toc112596670)

[**Residuals diagnostics** 11](#_Toc112596671)

[***Figure S1*. Residuals plots from the fully adjusted model with migrants grouped by generational status.** 11](#_Toc112596672)

[***Figure S2.* Residuals plots from the fully adjusted model with migrants grouped by region of origin.** 11](#_Toc112596673)

[***Table S8.* Predicted probabilities of first-episode psychosis by child maltreatment and migrant generational status.** 12](#_Toc112596674)

[***Table S9.* Predicted probabilities of first-episode psychosis by child maltreatment and Western/non-Western migrant status.** 13](#_Toc112596675)

[**Sensitivity analyses** 14](#_Toc112596676)

[***Table S8.* Sensitivity analyses of the association between first-episode psychosis and child maltreatment by migrant status.** 14](#_Toc112596677)

[**Acknowledgments** 15](#_Toc112596678)

[**Author contributions** 17](#_Toc112596679)

[**Conflicts of Interests** 17](#_Toc112596680)

[**Role of the funding source** 17](#_Toc112596681)

[**Supplemental References** 18](#_Toc112596682)

# **Missing data**

The proportion of participants with missing data was generally low and ranged from none on gender and country to 269 (13.5%) on parental psychosis. Complete data were available for 1,502 participants (75.4%). Cases were more likely than controls to be missing on cannabis use (n=30, 3.5% vs n=11, 1.0%; χ2=16.0; p=0.001), education (n=20, 2.4% vs n=7, 0.6%; χ2=11.1; p<0.001), parental social class (n=98, 11.5% vs n=63, 5.5%; χ2=23.8; p<0.001), EA (n=74, 8.7% vs n=10, 0.9%; χ2=74.1; p<0.001), EN (n=74, 8.7% vs n=10, 0.9%; χ2=74.1; p<0.001), PA (n=72, 8.5% vs n=10, 0.9%; χ2=71.3; p<0.001), PN (n=72, 8.5% vs n=10, 0.9%; χ2=71.3; p<0.001), and SA (n=76, 9.0% vs n=11, 1.0%; χ2=74.4; p<0.001 ). Missing values were imputed via the “missRanger” package of R (Stekhoven and Bühlmann, 2021), which is based on the algorithm of “missForest” (Stekhoven and Bühlmann, 2012), allowing to handle missing variables using random forest models (Breiman, 2001; Liaw and Wiener, 2002). This method was found to offer good performance and the lowest error when compared to other popular techniques, such as multiple imputation by chained equations (MICE) and nearest neighbour estimation (Waljee et al., 2013). Following imputation, we computed out-of-bag errors for each variable as a measure of accuracy. Values closer to 0 indicate a better performance. In our study, errors were comprised between 0.000 and 0.151. TableS1 shows distribution of exposures and covariates along with the missing proportion in the case-control sample. Out-of-bag errors are reported in the Table for all variables which required imputation.

# ***Table S1.* Distribution of exposures and covariates in the non-imputed case-control sample.**

|  |  | **Total sample (N=1,991)** | | |  |
| --- | --- | --- | --- | --- | --- |
|  |  | **Controls (N=1,142)** | **Cases (N=849)** | **χ^2^/t (p)** | **OOB** |
| **Gender** | Males | 604 (52.9%) | 318 (37.5%) | **46.7 (p<0.001)^a^** | **-** |
|  | Females | 538 (47.1%) | 531 (62.5%) |  |  |
|  | Missing | - | - |  |  |
| **Age** | Mean (SD) | 36.6 (13.1) | 31.1 (10.4) | **10.2 (<0.001)^b^** | **-** |
|  | Missing | - | - |  |  |
| **Migrant generational status** | Reference population | 769 (67.3%) | 478 (56.3%) | **28.7 (<0.001)^a^** | **-** |
|  | First generation | 214 (18.7%) | 237 (27.9%) |  |  |
|  | Further generation | 159 (13.9%) | 134 (15.8%) |  |  |
|  | Missing | - | - |  |  |
| **Migrant group** | Reference population | 769 (67.3%) | 478 (56.3%) | **35.0 (<0.001)^a^** | **-** |
|  | Western migrants | 131 (11.5%) | 92 (10.8%) |  |  |
|  | Non-Western migrants | 242 (21.2%) | 279 (32.9%) |  |  |
|  | Missing | - | - |  |  |
| **Education (years)** | Mean (SD) | 15.4 (3.9) | 13.8 (3.9) | **9.4 (<0.001)^b^** | 0.128 |
|  | Missing | 7 (0.9%) | 20 (2.4%) |  |  |
| **Parental social class** | Professional | 389 (34.1%) | 247 (29.1%) | **13.3 (0.004)^a^** | 0.013 |
|  | Intermediate | 277 (24.3%) | 196 (23.1%) |  |  |
|  | Working class | 468 (41.0%) | 389 (45.8%) |  |  |
|  | Long-term unemployed | 8 (0.7%) | 17 (2.0%) |  |  |
|  | Missing | 63 (5.5%) | 98 (11.5%) |  |  |
| **Parental psychosis** | No | 1,123 (98.3%) | 792 (93.3%) | **33.8 (<0.001)^a^** | 0.000 |
|  | Yes | 19 (1.7%) | 57 (6.7%) |  |  |
|  | Missing | 154 (13.5%) | 115 (13.5%) |  |  |
| **Parental mental illness** | No | 885 (77.5%) | 574 (67.6%) | **24.3 (<0.001)^a^** | 0.000 |
|  | Yes | 257 (22.5%) | 275 (32.4%) |  |  |
|  | Missing | 147 (12.9%) | 104 (12.2%) |  |  |
| **Cannabis use** | No | 1,006 (88.1%) | 656 (77.3%) | **41.4 (<0.001)^a^** | 0.000 |
|  | Yes | 136 (11.9%) | 193 (22.7%) |  |  |
|  | Missing | 11 (1.0%) | 30 (3.5%) |  |  |
| **Emotional Abuse** | <10 | 953 (83.5%) | 541 (63.7%) | **101.2(<0.001)^a^** | 0.079 |
|  | ≥10 | 189 (16.5%) | 308 (36.3%) |  |  |
|  | Missing | 10 (0.9%) | 74 (8.7%) |  |  |
| **Emotional Neglect** | <15 | 999 (87.5%) | 615 (72.4%) | **71.8 (<0.001)^a^** | 0.077 |
|  | ≥15 | 143 (12.5%) | 234 (27.6%) |  |  |
|  | Missing | 10 (0.9%) | 74 (8.7%) |  |  |
| **Physical Abuse** | <8 | 1,018 (89.1%) | 612 (72.1%) | **95.4 (<0.001)^a^** | 0.105 |
|  | ≥8 | 124 (10.9%) | 237 (27.9%) |  |  |
|  | Missing | 10 (0.9%) | 72 (8.5%) |  |  |
| **Physical Neglect** | <8 | 891 (78.0%) | 458 (53.9%) | **129.2 (<0.001)^a^** | 0.107 |
|  | ≥8 | 251 (22.0%) | 391 (46.1%) |  |  |
|  | Missing | 10 (0.9%) | 72 (8.5%) |  |  |
| **Sexual Abuse** | <8 | 1,058 (92.6%) | 702 (82.7%) | **47.1 (<0.001)^a^** | 0.151 |
|  | ≥8 | 84 (7.4%) | 147 (17.3%) |  |  |
|  | Missing | 11 (1.0%) | 76 (9.0%) |  |  |

^a^Pearson's chi-squared test, ^b^t-student’s test, SD: standard deviation. OOB: out-of-bag error.

**Childhood Trauma Questionnaire scoring**

The Childhood Trauma Questionnaire (CTQ) is a standardized 28-item self-report inventory that retrospectively assesses the severity of five subtypes of childhood trauma along with participants’ tendency to underreport maltreatment (Bernstein *et al.*, 2003). It consists of five items for each subtype of trauma (emotional abuse, emotional neglect, physical abuse, physical neglect, and sexual abuse). Every item is rated on a 5-point Likert-type scale (1=never true, 5=very often true). For each subtype, fixed threshold values allow to categorize trauma severity as: none to minimal, slight to moderate, moderate to severe, severe to extreme (Bernstein *et al.*, 2003). Walker et al. (Walker *et al.*, 1999) have proposed another procedure to obtain binary measures of trauma subtypes with excellent sensitivity and specificity (≥0.85). Table S2 compares the proposed CTQ scorings.

**Table S2. Proposed scorings of Childhood Trauma Questionnaire.**

|  | **Bernstein et al. (2003)** | | | | **Walker et al. (1999)** |
| --- | --- | --- | --- | --- | --- |
|  | **None/minimal** | **Slight/moderate** | **Moderate/severe** | **Severe/extreme** |  |
| **Emotional Abuse** | 5-8 | 9-12 | 13-15 | 16-25 | 10-25 |
| **Emotional Neglect** | 5-9 | 10-14 | 15-17 | 18-25 | 15-25 |
| **Physical Abuse** | 5-7 | 8-9 | 10-12 | 13-25 | 8-25 |
| **Physical Neglect** | 5-7 | 8-9 | 10-12 | 13-25 | 8-25 |
| **Sexual Abuse** | 5 | 6-7 | 8-12 | 13-25 | 8-25 |

# ***Table S3.* Distribution of exposures and covariates by case-control and migrant generational status.**

|  | **Majority (N=1,247)** | | | **First-generation migrants (N=451)** | | | **Further-generation migrants (N=293)** | | | **Total sample (N=1,991)** | | |
| --- | --- | --- | --- | --- | --- | --- | --- | --- | --- | --- | --- | --- |
|  | **Controls**  **N (%)** | **Cases**  **N (%)** | **χ^2^/t**  **(p)** | **Controls**  **N (%)** | **Cases**  **N (%)** | **χ^2^/t**  **(p)** | **Controls**  **N (%)** | **Cases**  **N (%)** | **χ^2^/t**  **(p)** | **Controls** | **Cases** | **χ^2^/t**  **(p)** |
| **Gender** |  |  | **22.2**  **(<0.001)^a^** |  |  | **18.6**  **(<0.001)^a^** |  |  | **7.0**  **(<0.001)^a^** |  |  | **46.7**  **(p<0.001)^a^** |
| Males | 366 (52.4%) | 293 (61.3%) |  | 92 (43.0%) | 150 (63.3%) |  | 80 (50.3%) | 88 (65.7%) |  | 538 (47.1%) | 531 (62.5%) |  |
| Females | 403 (47.6%) | 185 (38.7%) |  | 122 (57.0%) | 87 (36.7%) |  | 79 (49.7%) | 46 (34.3%) |  | 604 (52.9%) | 318 (37.5%) |  |
| **Age**^b^ | 37.0  (13.4) | 32.2  (11.2) | **6.7**  **(<0.001)^c^** | 37.1  (12.0) | 31.1  (8.9) | **6.1**  **(<0.001)^c^** | 33.9  (13.0) | 27.2  (8.7) | **5.1**  **(<0.001)^c^** | 36.6  (13.1) | 31.1  (10.4) | **10.2**  **(<0.001)^c^** |
| **Education** | 15.3  (3.8) | 13.6  (4.1) | **7.2**  **(<0.001)^c^** | 15.7  (4.3) | 13.9  (3.9) | **4.6**  **(<0.001)^c^** | 15.7  (3.3) | 14.0  (3.1) | **4.7**  **(<0.001)^c^** | 15.4  (3.9) | 13.8  (3.9) | **9.4**  **(<0.001)^c^** |
| **Parental social class** |  |  | 5.2  (0.150)^d^ |  |  | 6.2  (0.098)^d^ |  |  | **13.3**  **(0.003)^d^** |  |  | **13.3**  **(0.004)^a^** |
| Professional | 234 (30.4%) | 135 (28.2%) |  | 89 (41.6%) | 83 (35.0%) |  | 66 (41.5%) | 29 (21.6%) |  | 389 (34.1%) | 247 (29.1%) |  |
| Intermediate | 204 (26.5%) | 114 (23.8%) |  | 50 (23.4%) | 56 (23.6%) |  | 23 (14.5%) | 26 (19.4%) |  | 277 (24.3%) | 196 (23.1%) |  |
| Working class | 327 (42.5%) | 222 (46.4%) |  | 75 (35.0%) | 93 (39.2%) |  | 66 (41.5%) | 74 (55.2%) |  | 468 (41.0%) | 389 (45.8%) |  |
| Long-term unemployed | 4 (0.5%) | 7 (1.5%) |  | 0 (0.0%) | 5 (2.1%) |  | 4 (2.5%) | 5 (3.7%) |  | 8 (0.7%) | 17 (2.0%) |  |
| **Parental psychosis** |  |  | **13.6**  **(<0.001)^a^** |  |  | **5.5**  **(0.019)^a^** |  |  | **15.7**  **(<0.001)^a^** |  |  | **33.8**  **(<0.001)^a^** |
| No | 755 (98.2%) | 451 (94.4%) |  | 211 (98.6%) | 224 (94.5%) |  | 157 (98.7%) | 117 (87.3%) |  | 1,123 (98.3%) | 792 (93.3%) |  |
| Yes | 14 (1.8%) | 27 (5.6%) |  | 3 (1.4%) | 13 (5.5%) |  | 2 (1.3%) | 17 (12.7%) |  | 19 (1.7%) | 57 (6.7%) |  |
| **Parental mental illness** |  |  | **21.4**  **(<0.001)^a^** |  |  | 2.0  (0.153)^a^ |  |  | **5.7**  **(0.017)^a^** |  |  | **24.3**  **(<0.001)^a^** |
| No | 590 (76.7%) | 309 (64.6%) |  | 176 (82.2%) | 182 (76.8%) |  | 119 (74.8%) | 83 (61.9%) |  | 885 (77.5%) | 574 (67.6%) |  |
| Yes | 179 (23.3%) | 169 (35.4%) |  | 38 (17.8%) | 55 (23.2%) |  | 40 (25.2%) | 51 (38.1%) |  | 257 (22.5%) | 275 (32.4%) |  |
| **Cannabis use** |  |  | **15.1**  **(<0.001)^a^** |  |  | **19.5**  **(<0.001)^a^** |  |  | **19.5**  **(<0.001)^a^** |  |  | **41.4**  **(<0.001)^a^** |
| No | 682 (88.7%) | 386 (80.8%) |  | 192 (89.7%) | 174 (73.4%) |  | 132 (83.0%) | 96 (71.6%) |  | 1,006 (88.1%) | 656 (77.3%) |  |
| Yes | 87 (11.3%) | 92 (19.2%) |  | 22 (10.3%) | 63 (26.6%) |  | 27 (17.0%) | 38 (28.4%) |  | 136 (11.9%) | 193 (22.7%) |  |
| **Child maltreatment** |  |  | **90.8**  **(<0.001)^a^** |  |  | **30.2**  **(<0.001)^a^** |  |  | **25.1**  **(<0.001)^a^** |  |  | **159.6**  **(<0.001)^a^** |
| No | 655 (85.2%) | 294 (61.5%) |  | 158 (73.8%) | 115 (48.5%) |  | 123 (77.4%) | 66 (49.3%) |  | 936 (82.0%) | 475 (55.9%) |  |
| Yes | 114 (14.8%) | 478 (38.5%) |  | 56 (26.2%) | 122 (51.5%) |  | 36 (22.6%) | 68 (50.7%) |  | 206 (18.0%) | 374 (44.1%) |  |
| **Emotional Abuse** |  |  | **58.7**  **(<0.001)^a^** |  |  | **18.6**  **(<0.001)^a^** |  |  | **15.2**  **(<0.001)^a^** |  |  | **101.2**  **(<0.001)^a^** |
| No | 661 (86.0%) | 324 (67.8%) |  | 167 (78.0%) | 140 (59.1%) |  | 125 (78.6%) | 77 (57.5%) |  | 953 (83.5%) | 541 (63.7%) |  |
| Yes | 108 (14.0%) | 154 (32.2%) |  | 47 (22.0%) | 97 (40.9%) |  | 34 (21.4%) | 57 (42.5%) |  | 189 (16.5%) | 308 (36.3%) |  |
| **Emotional Neglect** |  |  | **35.9**  **(<0.001)^a^** |  |  | **14.4**  **(<0.001)^a^** |  |  | **17.5**  **(<0.001)^a^** |  |  | **71.8**  **(<0.001)^a^** |
| No | 678 (88.2%) | 359 (75.1%) |  | 182 (85.0%) | 166 (70.0%) |  | 139 (87.4%) | 90 (67.2%) |  | 999 (87.5%) | 615 (72.4%) |  |
| Yes | 91 (11.8%) | 119 (24.9%) |  | 32 (15.0%) | 71 (30.0%) |  | 20 (12.6%) | 44 (32.8%) |  | 143 (12.5%) | 234 (27.6%) |  |
| **Physical Abuse** |  |  | **40.0**  **(<0.001)^a^** |  |  | **20.7**  **(<0.001)^a^** |  |  | **16.3**  **(<0.001)^a^** |  |  | **95.4**  **(<0.001)^a^** |
| No | 723 (94.0%) | 396 (82.8%) |  | 166 (77.6%) | 136 (57.4%) |  | 129 (81.1%) | 80 (59.7%) |  | 1,018 (89.1%) | 612 (72.1%) |  |
| Yes | 46 (6.0%) | 82 (17.2%) |  | 48 (22.4%) | 101 (42.6%) |  | 30 (18.9%) | 54 (40.3%) |  | 124 (10.9%) | 237 (27.9%) |  |
| **Physical Neglect** |  |  | **46.3**  **(<0.001)^a^** |  |  | **26.5**  **(<0.001)^a^** |  |  | **48.2**  **(<0.001)^a^** |  |  | **129.2**  **(<0.001)^a^** |
| No | 621 (80.8%) | 303 (63.4%) |  | 143 (66.8%) | 101 (42.6%) |  | 127 (79.9%) | 54 (40.3%) |  | 891 (78.0%) | 458 (53.9%) |  |
| Yes | 148 (19.2%) | 175 (36.6%) |  | 71 (33.2%) | 136 (57.4%) |  | 32 (20.1%) | 80 (59.7%) |  | 251 (22.0%) | 391 (46.1%) |  |
| **Sexual Abuse** |  |  | **10.4**  **(<0.001)^a^** |  |  | **18.7**  **(<0.001)^a^** |  |  | **10.0**  **(<0.001)^a^** |  |  | **47.1**  **(<0.001)^a^** |
| No | 724 (94.1%) | 426 (89.1%) |  | 192 (89.7%) | 175 (73.8%) |  | 142 (89.3%) | 101 (75.4%) |  | 1,058 (92.6%) | 702 (82.7%) |  |
| Yes | 45 (5.9%) | 52 (10.9%) |  | 22 (10.3%) | 62 (26.2%) |  | 17 (10.7%) | 33 (24.6%) |  | 84 (7.4%) | 147 (17.3%) |  |

^a^Pearson's chi-squared test ^b^mean (SD) ^c^t-student’s test ^d^Fisher’s exact test

# ***Table S4.* Distribution exposures and covariates by case-control and migrant group.**

|  | **Majority (N=1,247)** | | | **Western migrants (N=223)** | | | **Non-western migrants (N=521)** | | | **Total sample (N=1,991)** | | |
| --- | --- | --- | --- | --- | --- | --- | --- | --- | --- | --- | --- | --- |
|  | **Controls**  **N (%)** | **Cases**  **N (%)** | **χ^2^/t**  **(p)** | **Controls**  **N (%)** | **Cases**  **N (%)** | **χ^2^/t**  **(p)** | **Controls**  **N (%)** | **Cases**  **N (%)** | **χ^2^/t**  **(p)** | **Controls** | **Cases** | **χ^2^/t**  **(p)** |
| **Gender** |  |  | **22.2**  **(<0.001)^a^** |  |  | **4.5**  **(0.034)^a^** |  |  | **18.8**  **(<0.001)^a^** |  |  | **46.7**  **(p<0.001)^a^** |
| Males | 366 (47.6%) | 293 (61.3%) |  | 58 (44.3%) | 54 (58.7%) |  | 114 (47.1%) | 184 (65.9%) |  | 604 (52.9%) | 318 (37.5%) |  |
| Females | 403 (52.4%) | 185 (38.7%) |  | 73 (55.7%) | 38 (41.3%) |  | 128 (52.9%) | 95 (34.1%) |  | 538 (47.1%) | 531 (62.5%) |  |
| **Age**^b^ | 37.0  (13.4) | 32.2  (11.2) | **6.7**  **(<0.001)^c^** | 37.1  (12.0) | 31.1  (8.9) | **6.1**  **(<0.001)^c^** | 33.9  (13.0) | 27.2  (8.7) | **5.1**  **(<0.001)^c^** | 36.6  (13.1) | 31.1  (10.4) | **10.2**  **(<0.001)^c^** |
| **Education** | 15.3  (3.8) | 13.6  (4.1) | **7.2**  **(<0.001)^c^** | 15.7  (4.3) | 13.9  (3.9) | **4.6**  **(<0.001)^c^** | 15.7  (3.3) | 14.0  (3.1) | **4.7**  **(<0.001)^c^** | 15.4  (3.9) | 13.8  (3.9) | **9.4**  **(<0.001)^c^** |
| **Parental social class** |  |  | 5.2  (0.150)^d^ |  |  | **10.4**  **(0.011)^d^** |  |  | 5.3  (0.150)^d^ |  |  | **13.3**  **(0.004)^a^** |
| Professional | 234 (30.4%) | 135 (28.2%) |  | 57 (43.5%) | 26 (28.3%) |  | 98 (40.5%) | 86 (30.8%) |  | 389 (34.1%) | 247 (29.1%) |  |
| Intermediate | 204 (26.5%) | 114 (23.8%) |  | 24 (18.3%) | 16 (17.4%) |  | 49 (20.2%) | 66 (23.7%) |  | 277 (24.3%) | 196 (23.1%) |  |
| Working class | 327 (42.5%) | 222 (46.4%) |  | 50 (38.2%) | 46 (50.0%) |  | 91 (37.6%) | 121 (43.4%) |  | 468 (41.0%) | 389 (45.8%) |  |
| Long-term unemployed | 4 (0.5%) | 7 (1.5%) |  | 0 (0.0%) | 4 (4.3%) |  | 4 (1.7%) | 6 (2.2%) |  | 8 (0.7%) | 17 (2.0%) |  |
| **Parental psychosis** |  |  | **13.6**  **(<0.001)^a^** |  |  | **11.7**  **(0.001)^d^** |  |  | **9.0**  **(0.003)^a^** |  |  | **33.8**  **(<0.001)^a^** |
| No | 755 (98.2%) | 451 (94.4%) |  | 130 (99.2%) | 82 (89.1%) |  | 238 (98.3%) | 259 (92.8%) |  | 1,123 (98.3%) | 792 (93.3%) |  |
| Yes | 14 (1.8%) | 27 (5.6%) |  | 1 (0.8%) | 10 (10.9%) |  | 4 (1.7%) | 20 (7.2%) |  | 19 (1.7%) | 57 (6.7%) |  |
| **Parental mental illness** |  |  | **21.4**  **(<0.001)^a^** |  |  | **4.2**  **(0.040)^a^** |  |  | 3.5  (0.061)^a^ |  |  | **24.3**  **(<0.001)^a^** |
| No | 590 (76.7%) | 309 (64.6%) |  | 98 (74.8%) | 57 (62.0%) |  | 197 (81.4%) | 208 (74.6%) |  | 885 (77.5%) | 574 (67.6%) |  |
| Yes | 179 (23.3%) | 169 (35.4%) |  | 33 (25.2%) | 35 (38.0%) |  | 45 (18.6%) | 71 (25.4%) |  | 257 (22.5%) | 275 (32.4%) |  |
| **Cannabis use** |  |  | **15.1**  **(<0.001)^a^** |  |  | **3.9**  **(<0.050)^a^** |  |  | **19.1**  **(<0.001)^a^** |  |  | **41.4**  **(<0.001)^a^** |
| No | 682 (88.7%) | 386 (80.8%) |  | 112 (85.5%) | 69 (75.0%) |  | 212 (87.6%) | 201 (72.0%) |  | 1,006 (88.1%) | 656 (77.3%) |  |
| Yes | 87 (11.3%) | 92 (19.2%) |  | 19 (14.5%) | 23 (25.0%) |  | 30 (12.4%) | 78 (28.0%) |  | 136 (11.9%) | 193 (22.7%) |  |
| **Child maltreatment (CTQ)^b^** |  |  | **90.8**  **(<0.001)^a^** |  |  | **16.2**  **(0.001)^a^** |  |  | **37.3**  **(<0.001)^a^** |  |  | **159.6**  **(<0.001)^a^** |
| No | 655 (85.2%) | 294 (61.5%) |  | 102 (77.9%) | 48 (52.2%) |  | 179 (74.0%) | 133 (47.7%) |  | 936 (82.0%) | 475 (55.9%) |  |
| Yes | 114 (14.8%) | 478 (38.5%) |  | 29 (22.1%) | 44 (47.8%) |  | 63 (26.0%) | 146 (52.3%) |  | 206 (18.0%) | 374 (44.1%) |  |
| **Emotional Abuse** |  |  | **58.7**  **(<0.001)^a^** |  |  | **14.5**  **(<0.001)^a^** |  |  | **19.4**  **(<0.001)^a^** |  |  | **101.2**  **(<0.001)^a^** |
| No | 661 (86.0%) | 324 (67.8%) |  | 105 (80.2%) | 52 (56.5%) |  | 187 (77.3%) | 165 (59.1%) |  | 953 (83.5%) | 541 (63.7%) |  |
| Yes | 108 (14.0%) | 154 (32.2%) |  | 26 (19.8%) | 40 (43.5%) |  | 55 (22.7%) | 114 (40.9%) |  | 189 (16.5%) | 308 (36.3%) |  |
| **Emotional Neglect** |  |  | **35.9**  **(<0.001)^a^** |  |  | **12.5**  **(<0.001)^a^** |  |  | **18.4**  **(<0.001)^a^** |  |  | **71.8**  **(<0.001)^a^** |
| No | 678 (88.2%) | 359 (75.1%) |  | 115 (87.8%) | 63 (68.5%) |  | 206 (85.1%) | 193 (69.2%) |  | 999 (87.5%) | 615 (72.4%) |  |
| Yes | 91 (11.8%) | 119 (24.9%) |  | 16 (12.2%) | 29 (31.5%) |  | 36 (14.9%) | 86 (30.8%) |  | 143 (12.5%) | 234 (27.6%) |  |
| **Physical Abuse** |  |  | **40.0**  **(<0.001)^a^** |  |  | **7.2**  **(0.007)^a^** |  |  | **25.8**  **(<0.001)^a^** |  |  | **95.4**  **(<0.001)^a^** |
| No | 723 (94.0%) | 396 (82.8%) |  | 113 (86.3%) | 66 (71.7%) |  | 182 (75.2%) | 150 (53.8%) |  | 1,018 (89.1%) | 612 (72.1%) |  |
| Yes | 46 (6.0%) | 82 (17.2%) |  | 18 (13.7%) | 26 (28.3%) |  | 60 (24.8%) | 129 (46.2%) |  | 124 (10.9%) | 237 (27.9%) |  |
| **Physical Neglect** |  |  | **46.3**  **(<0.001)^a^** |  |  | **26.0**  **(<0.001)^a^** |  |  | **42.6**  **(<0.001)^a^** |  |  | **129.2**  **(<0.001)^a^** |
| No | 621 (80.8%) | 303 (63.4%) |  | 102 (77.9%) | 41 (44.6%) |  | 168 (69.4%) | 114 (40.9%) |  | 891 (78.0%) | 458 (53.9%) |  |
| Yes | 148 (19.2%) | 175 (36.6%) |  | 29 (22.1%) | 51 (55.4%) |  | 74 (30.6%) | 165 (59.1%) |  | 251 (22.0%) | 391 (46.1%) |  |
| **Sexual Abuse** |  |  | **10.4**  **(<0.001)^a^** |  |  | **9.4**  **(0.002)^a^** |  |  | **17.6**  **(<0.001)^a^** |  |  | **47.1**  **(<0.001)^a^** |
| No | 724 (94.1%) | 426 (89.1%) |  | 122 (93.1%) | 73 (79.3%) |  | 212 (87.6%) | 203 (72.8%) |  | 1,058 (92.6%) | 702 (82.7%) |  |
| Yes | 45 (5.9%) | 52 (10.9%) |  | 9 (6.9%) | 19 (20.7%) |  | 30 (12.4%) | 76 (27.2%) |  | 84 (7.4%) | 147 (17.3%) |  |

^a^Pearson's chi-squared test ^b^mean (SD) ^c^t-student’s test ^d^Fisher’s exact test

# **Correlations between child maltreatment subtypes**

Correlation between child maltreatment subtypes was assessed using Pearson’s test. All measures of child maltreatment were significantly and positively correlated (p<0.001). The correlation matrix is shown in Table S5.

# ***Table S5.* Correlation matrix of child maltreatment subtypes.**

| Variable | 1. | 2. | 3. | 4. | 5. |
| --- | --- | --- | --- | --- | --- |
| 1. | 1 |  |  |  |  |
| 2. | .59 | 1 |  |  |  |
| 3. | .63 | .41 | 1 |  |  |
| 4. | .48 | .60 | .46 | 1 |  |
| 5. | .38 | .25 | .43 | .29 | 1 |
| 1. Emotional Abuse  2. Emotional Neglect  3. Physical Abuse  4. Physical Neglect  5. Sexual Abuse | | | | | |

# **Correlations between exposures and confounders**

Table S5 and Table S6 detail the polychoric correlations between the various exposures and confounders in our sample (as a representation of the general population). We did not detect any strong correlation (coefficient of at least ± 0.6). The only moderate correlation (coefficient of at least ± 0.4) was a negative correlation between age and cannabis use. Weak correlations (coefficient of at least ± 0.2) were observed between sex and cannabis use, and between child maltreatment and both definitions migrant status (all positive). We further estimated the Variance Inflation Factor (VIF) for each independent variable along with the mean VIF for each model. All VIFs were close to the value of 1, with the mean VIF being 1.19 in the model with migrants defined based on generational status and 1.18 in the model with migrants defined based on area of origin.

# ***Table S6.* Correlation matrix of predictors included in the model with migrants defined based on generational status.**

| Variable | 1. | 2. | 3. | 4. | 5. | 6. | 7. | 8. |
| --- | --- | --- | --- | --- | --- | --- | --- | --- |
| 1. | 1 |  |  |  |  |  |  |  |
| 2. | -.12 | 1 |  |  |  |  |  |  |
| 3. | -.01 | -.05 | 1 |  |  |  |  |  |
| 4. | -.40 | .36 | -.02 | 1 |  |  |  |  |
| 5. | .06 | .01 | -.17 | .05 | 1 |  |  |  |
| 6. | -.13 | .05 | -.00 | .10 | .16 | 1 |  |  |
| 7. | -.03 | .00 | -.15 | .16 | .05 | .19 | 1 |  |
| 8. | -.15 | .04 | .03 | .14 | .04 | .13 | .22 | 1 |
| 1. Age  2. Gender  3. Education  4. Cannabis use  5. Parental social class  6. Parental psychosis  7. Child maltreatment  8. Migrant generation | | | | | | | |  |

# ***Table S7.* Correlation matrix of predictors included in the model with migrants defined based on area of origin.**

| Variable | 1. | 2. | 3. | 4. | 5. | 6. | 7. | 8. |
| --- | --- | --- | --- | --- | --- | --- | --- | --- |
| 1. | 1 |  |  |  |  |  |  |  |
| 2. | -.12 | 1 |  |  |  |  |  |  |
| 3. | -.01 | -.05 | 1 |  |  |  |  |  |
| 4. | -.40 | .36 | -.02 | 1 |  |  |  |  |
| 5. | .06 | .01 | -.17 | .05 | 1 |  |  |  |
| 6. | -.13 | .05 | -.00 | .10 | .16 | 1 |  |  |
| 7. | -.03 | .00 | -.15 | .16 | .05 | .19 | 1 |  |
| 8. | -.14 | .05 | .01 | .14 | .00 | .09 | .25 | 1 |
| 1. Age  2. Gender  3. Education  4. Cannabis use  5. Parental social class  6. Parental psychosis  7. Child maltreatment  8. Migrant group | | | | | | | | |

# **Residuals diagnostics**

The R package “DHARMa” (Hartig, 2022) was used to check the distribution of the residuals of the fully adjusted mixed-effect logistic regression models. Figures (Figure S1-S2) below show that residuals were normally distributed. The estimated dispersion was 0.99 for both the model including migrants grouped by generational status and the one including migrants grouped by region of origin.

# ***Figure S1*. Residuals plots from the fully adjusted model with migrants grouped by generational status.**

# ***Figure S2.* Residuals plots from the fully adjusted model with migrants grouped by region of origin.**

# ***Table S8.* Predicted probabilities of first-episode psychosis by child maltreatment and migrant generational status.**

| **Child maltreatment score^†^** |  |  | **Predicted probability** | **Standard error** | **p-value** |
| --- | --- | --- | --- | --- | --- |
| -1 |  | Reference | .258 | .028 | <0.001 |
|  |  | First-generation | .420 | .043 | <0.001 |
|  |  | Further-generation | .288 | .043 | <0.001 |
| 0 |  | Reference | .421 | .031 | <0.001 |
|  |  | First-generation | .509 | .037 | <0.001 |
|  |  | Further-generation | .400 | .041 | <0.001 |
| 1 |  | Reference | .601 | .037 | <0.001 |
|  |  | First-generation | .596 | .040 | <0.001 |
|  |  | Further-generation | .523 | .048 | <0.001 |
| 2 |  | Reference | .759 | .039 | <0.001 |
|  |  | First-generation | .679 | .048 | <0.001 |
|  |  | Further-generation | .643 | .060 | <0.001 |
| 3 |  | Reference | .872 | .032 | <0.001 |
|  |  | First-generation | .753 | .054 | <0.001 |
|  |  | Further-generation | .748 | .066 | <0.001 |
| 4 |  | Reference | .938 | .022 | <0.001 |
|  |  | First-generation | .815 | .057 | <0.001 |
|  |  | Further-generation | .833 | .064 | <0.001 |

^†^Standardized mean CTQ score.

***Table S9.* Predicted probabilities of first-episode psychosis by child maltreatment and Western/non-Western migrant status.**

| **Child maltreatment score^†^** |  |  | **Predicted probability** | **Standard error** | **p-value** |
| --- | --- | --- | --- | --- | --- |
| -1 |  | Reference | .257 | .028 | <0.001 |
|  |  | Western | .299 | .049 | <0.001 |
|  |  | Non-Western | .399 | .042 | <0.001 |
| 0 |  | Reference | .421 | .032 | <0.001 |
|  |  | Western | .426 | .044 | <0.001 |
|  |  | Non-Western | .490 | .038 | <0.001 |
| 1 |  | Reference | .601 | .037 | <0.001 |
|  |  | Western | .563 | .053 | <0.001 |
|  |  | Non-Western | .586 | .040 | <0.001 |
| 2 |  | Reference | .760 | .039 | <0.001 |
|  |  | Western | .692 | .066 | <0.001 |
|  |  | Non-Western | .670 | .046 | <0.001 |
| 3 |  | Reference | .872 | .032 | <0.001 |
|  |  | Western | .798 | .070 | <0.001 |
|  |  | Non-Western | .747 | .051 | <0.001 |
| 4 |  | Reference | .939 | .022 | <0.001 |
|  |  | Western | .877 | .063 | <0.001 |
|  |  | Non-Western | .813 | .053 | <0.001 |

^†^Standardized mean CTQ score

# **Sensitivity analyses**

Sensitivity analyses were conducted on the complete-case sample only for the main outcomes. Magnitude and directions of associations were similar to imputed analyses (Table S8).

# ***Table S8.* Sensitivity analyses of the association between first-episode psychosis and child maltreatment by migrant status.**

|  |  |  | | **Unadjusted** | | **Adjusted^†^** | | **Migrant X CTQ^††^** | | **PAF (95%CI)** |
| --- | --- | --- | --- | --- | --- | --- | --- | --- | --- | --- |
|  |  | N controls (%) | N cases (%) | OR (95%CI) | p | OR (95%CI) | p | OR (95%CI) | p |  |
| *By generational status* | Reference | 617 (67.1%) | 348 (59.7%) | Ref. | - | Ref. | - | **2.35 (1.93-2.86)** | <0.001 | 30.0% (26.8-33.0%) |
|  | First generation | 172 (18.7%) | 147 (25.2%) | **1.74 (1.32-2.30)** | <0.001 | **1.47 (1.08-2.01)** | 0.015 | **1.58 (1.26-1.98)** | <0.001 | 40.5% (36.2-44.6%) |
|  | Further generation | 130 (14.2%) | 88 (15.1%) | **1.46 (1.04-2.03)** | 0.008 | 0.97 (0.67-1.40) | 0.861 | **1.76 (1.35-2.28)** | <0.001 | 34.7% (31.0-38.2%) |
| *By area of origin* | Reference | 617 (67.1%) | 348 (59.7%) | Ref. | - | Ref. | - | **2.34 (1.93-2.85)** | <0.001 | 30.0 (26.8-33.0%) |
|  | Western | 110 (12.0%) | 58 (10.0%) | 1.09 (0.76-1.57) | 0.642 | 0.91 (0.61-1.36) | 0.651 | **1.77 (1.30-2.42)** | <0.001 | 30.6 (27.3-33.7%) |
|  | Non-Western | 192 (20.9%) | 177 (30.3%) | **1.99 (1.50-2.63)** | <0.001 | **1.48 (1.08-2.01)** | 0.014 | **1.61 (1.31-1.98)** | <0.001 | 40.9% (36.5-45.0%) |

OR: odds ratio, 95%CI: 95% confidence interval, PAF: proportion of attributable fraction.

^†^Odds ratios were adjusted for age, gender, educational attainment, cannabis use, parental social class, and parental psychosis.

^††^Odds ratios were estimated by linear combination of the coefficients from the interaction model.

Likelihood-ratio test results: χ^2^=7.53, p=0.023 for the model using generational status; χ^2^=7.14; p=0.028 for the model using region of origin.

All models were mixed effect models accounting for clustering by site of recruitment (n=13).

# **Acknowledgments**

EU-GEI WP2 Group: The European Network of National Schizophrenia Networks Studying Gene-Environment Interactions (EU-GEI) WP2 Group members include : Kathryn Hubbard, MSc, Department of Health Service and Population Research, Institute of Psychiatry, King’s College London, De Crespigny Park, Denmark Hill, London, England; Stephanie Beards, PhD, Department of Health Service and Population Research, Institute of Psychiatry, King’s College London, De Crespigny Park, Denmark Hill, London, England; Pedro Cuadrado, MD, Villa de Vallecas Mental Health Department, Villa de Vallecas Mental Health Centre, Hospital Universitario Infanta Leonor/Hospital Virgen de la Torre, Madrid, Spain; José Juan Rodríguez Solano, MD, Puente de Vallecas Mental Health Department, Hospital Universitario Infanta Leonor/Hospital Virgen de la Torre, Centro de Salud Mental Puente de Vallecas, Madrid, Spain; Angel Carracedo, MD, PhD, Fundación Pública Galega de Medicina Xenómica, Hospital Clínico Universitario, Santiago de Compostela, Spain; Gonzalo López, PhD, Department of Child and Adolescent Psychiatry, Hospital General Universitario Gregorio Marañón, School of Medicine, Universidad Complutense, Investigación Sanitaria del Hospital Gregorio Marañón (Centro de Investigación Biomédica en Red de Salud Mental), Madrid, Spain; Silvia Amoretti, PhD, Department of Psychology and Psychiatry, Hospital Clinic, Institut d’Investigacions Biomèdiques August Pi i Sunyer, Centro de Investigación Biomédica en Red de Salud Mental, University of Barcelona, Barcelona, Spain; Eduardo J. Aguilar, MD, PhD, Department of Medicine, University of Valencia, INCLIVA, CIBERSAM, Valencia, Spain.; Paz Garcia-Portilla, MD, PhD, Department of Medicine, Psychiatry Area, School of Medicine, Universidad de Oviedo, Centro de Investigación Biomédica en Red de Salud Mental, Oviedo, Spain; Javier Costas, PhD, Fundación Pública Galega de Medicina Xenómica, Hospital Clínico Universitario, Santiago de Compostela, Spain; Estela Jiménez-López, MSc, Department of Psychiatry, Servicio de Psiquiatría Hospital “Virgen de la Luz,” Cuenca, Spain; Mario Matteis, MD, Department of Child and Adolescent Psychiatry, Hospital General Universitario Gregorio Marañón, School of Medicine, Universidad Complutense, Investigación Sanitaria del Hospital Gregorio Marañón (Centro de Investigación Biomédica en Red de Salud Mental), Madrid, Spain; Emiliano González, PhD, Department of Child and Adolescent Psychiatry, Hospital General Universitario Gregorio Marañón, School of Medicine, Universidad Complutense, Investigación Sanitaria del Hospital Gregorio Marañón (Centro de Investigación Biomédica en Red de Salud Mental), Madrid, Spain; Emilio Sánchez, MD, Department of Psychiatry, Hospital General Universitario Gregorio Marañón, School of Medicine, Universidad Complutense, Investigación Sanitaria del Hospital Gregorio Marañón (Centro de Investigación Biomédica en Red de Salud Mental), Madrid, Spain; Nathalie Franke, MSc, Department of Psychiatry, Early Psychosis Section, Academic Medical Centre, University of Amsterdam, Amsterdam, the Netherlands; Jean-Paul Selten, MD, PhD, Department of Psychiatry and Neuropsychology, School for Mental Health and Neuroscience, Maastricht University Medical Centre, Maastricht, the Netherlands, and Rivierduinen Institute for Mental Health Care, Leiden, the Netherlands; Fabian Termorshuizen, PhD, Department of Psychiatry and Neuropsychology, School for Mental Health and Neuroscience, South Limburg Mental Health Research and Teaching Network, Maastricht University Medical Centre, Maastricht, the Netherlands, and Rivierduinen Centre for Mental Health, Leiden, the Netherlands; Daniella van Dam, PhD, Department of Psychiatry, Early Psychosis Section, Academic Medical Centre, University of Amsterdam, Amsterdam, the Netherlands; Elles Messchaart, MSc, Rivierduinen Centre for Mental Health, Leiden, the Netherlands; Marion Leboyer, MD, PhD, Univ Paris Est Creteil (UPEC), AP-HP, Hôpitaux Universitaires « H. Mondor », DMU IMPACT, INSERM, IMRB, Fondation FondaMental, F-94010 Creteil, France.; Franck Schürhoff, MD, PhD, AP-HP, Univ Paris Est Creteil (UPEC), AP-HP, Hôpitaux Universitaires « H. Mondor », DMU IMPACT, INSERM, IMRB, Fondation FondaMental, F-94010 Creteil, France.; Stéphane Jamain, PhD, Univ Paris Est Creteil (UPEC), AP-HP, Hôpitaux Universitaires « H. Mondor », DMU IMPACT, INSERM, IMRB, Fondation FondaMental, F-94010 Creteil, France.; Flora Frijda, MSc, Etablissement Public de Santé Maison Blanche, Paris, France; Grégoire Baudin, MSc, Univ Paris Est Creteil (UPEC), AP-HP, Hôpitaux Universitaires « H. Mondor », DMU IMPACT, INSERM, IMRB, Fondation FondaMental, F-94010 Creteil, France.; Aziz Ferchiou, MD, AP-HP, Univ Paris Est Creteil (UPEC), AP-HP, Hôpitaux Universitaires « H. Mondor », DMU IMPACT, INSERM, IMRB, Fondation FondaMental, F-94010 Creteil, France.; Baptiste Pignon, MD, PhD, Univ Paris Est Creteil (UPEC), AP-HP, Hôpitaux Universitaires « H. Mondor », DMU IMPACT, INSERM, IMRB, Fondation FondaMental, F-94010 Creteil, France.; Jean-Romain Richard, MSc, Institut National de la Santé et de la Recherche Médicale, U955, Créteil, France, and Fondation Fondamental, Créteil, France; Thomas Charpeaud, MD, Fondation Fondamental, Créteil, France, CMP B CHU, Clermont Ferrand, France, and Université Clermont Auvergne, Clermont-Ferrand, France; Anne-Marie Tronche, MD, Fondation Fondamental, Créteil, France, CMP B CHU, Clermont Ferrand, France, and Université Clermont Auvergne, Clermont-Ferrand, France; Giovanna Marrazzo, MD, PhD, Unit of Psychiatry, “P. Giaccone” General Hospital, Palermo, Italy; Lucia Sideli, PhD, Department of Biomedicine, neurosciences, and advanced diagnostics, University of Palermo, , Via G. La Loggia 1, 90129 Palermo, Italy.; Crocettarachele Sartorio, PhD, Unit of Psychiatry, “P. Giaccone” General Hospital, Palermo, Italy;; Fabio Seminerio, PhD, Unit of Psychiatry, “P. Giaccone” General Hospital, Palermo, Italy; Camila Marcelino Loureiro, MD, Departamento de Neurociências e Ciencias do Comportamento, Faculdade de Medicina de Ribeirão Preto, Universidade de São Paulo, São Paulo, Brasil, and Núcleo de Pesquina em Saúde Mental Populacional, Universidade de São Paulo, São Paulo, Brasil; Rosana Shuhama, PhD, Departamento de Neurociências e Ciencias do Comportamento, Faculdade de Medicina de Ribeirão Preto, Universidade de São Paulo, São Paulo, Brasil, and Núcleo de Pesquina em Saúde Mental Populacional, Universidade de São Paulo, São Paulo, Brasil; Mirella Ruggeri, MD, PhD, Section of Psychiatry, Department of Neuroscience, Biomedicine and Movement, University of Verona, Verona, Italy; Chiara Bonetto, PhD, Section of Psychiatry, Department of Neuroscience, Biomedicine and Movement, University of Verona, Verona, Italy; Doriana Cristofalo, MA, Section of Psychiatry, Department of Neuroscience, Biomedicine and Movement, University of Verona, Verona, Italy; Marco Seri , Department of Medical and Surgical Sciences, Bologna University; Elena Bonora, Department of Medical and Surgical Sciences, Bologna University

# **Author contributions**

All the authors in the EU-GEI group collected or supervised the data collection. IT, JL, and GD were responsible for the conception and design of the study. IT, GD, CG-A, and ST cleaned and prepared the data for this paper analysis. GD and JL did the data analysis and wrote the findings in the initial manuscript. GD and JL contributed to the creation of the figures and tables. CM, RMM, MDF, DQ, and JK provided a careful statistical and methodological revision of the manuscript and contributed to the final draft. IT, GD, JL, RMM, CM, and ST contributed to the interpretation of the results. All authors had full access to all data (including statistical reports and tables) in the study and take responsibility for the integrity of the data and the accuracy of the data analysis.

# **Conflicts of Interests**

MDF reports personal fees from Janssen, outside the submitted work. RMM reports personal fees from Janssen, Lundbeck, Sunovion, and Otsuka, outside of the submitted work. PML reports personal fees from Janssen, Lundbeck, and Otsuka, outside of the submitted work. CA has been a consultant to or has received honoraria or grants from Acadia, Angelini, Gedeon Richter, Janssen Cilag, Lundbeck, Minerva, Otsuka, Roche, Sage, Servier, Shire, Schering Plough, Sumitomo Dainippon Pharma, Sunovion and Takeda. All authors declare no competing interests. JBK is supported by the National Institute for Health Research University College London Hospital Biomedical Research Centre and has received consultancy fees from Roche and the Health Services Executive, Ireland. MB has been a consultant for, received grant/research support and honoraria from, and been on the speakers/advisory board of ABBiotics, Adamed, Angelini, Casen Recordati, Janssen-Cilag, Menarini, Rovi and Takeda.

# **Role of the funding source**

Study funders contributed to the salaries of the research workers employed but did not participate in the study design, data analyses, data interpretation, or writing of the manuscript. All authors had full access to the study data and had final responsibility for the decision to submit for publication.

# **Supplemental References**

**Bernstein DP, Stein JA, Newcomb MD, Walker E, Pogge D, Ahluvalia T, Stokes J, Handelsman L, Medrano M, Desmond D, Zule W** (2003) Development and validation of a brief screening version of the Childhood Trauma Questionnaire. *Child abuse & neglect* **27**, 169–90.

**Breiman L** (2001) Random forests. *Machine Learning* **45**, 5–32.

**Hartig F** (2022) Residual Diagnostics for Hierarchical (Multi-Level / Mixed) Regression Models [R package DHARMa version 0.4.5]. Comprehensive R Archive Network (CRAN).

**Liaw A, Wiener M** (2002) Classification and Regression by RandomForest. *R News* **2**, 18–22.

**Stekhoven DJ, Bühlmann P** (2012) Missforest-Non-parametric missing value imputation for mixed-type data. Bioinformatics *Bioinformatics* **28**, 112–118.

**Stekhoven DJ, Bühlmann P** (2021) Fast Imputation of Missing Values [R package missRanger version 2.1.3]. Comprehensive R Archive Network (CRAN) *Bioinformatics* **28**, 112–118.

**Waljee AK, Mukherjee A, Singal AG, Zhang Y, Warren J, Balis U, Marrero J, Zhu J, Higgins PDR** (2013) Comparison of imputation methods for missing laboratory data in medicine. BMJ Publishing Group *BMJ Open* **3**.

**Walker EA, Gelfand A, Katon WJ, Koss MP, Von Korff M, Bernstein D, Russo J** (1999) Adult health status of women with histories of childhood abuse and neglect. Am J Med *American Journal of Medicine* **107**, 332–339.
